# Supplementary figures and images for: Optimisation of cell and ex vivo culture conditions to study vascular calcification
Source: PLoS One. 2020 Mar 6;15(3):e0230201. doi: 10.1371/journal.pone.0230201 (PMC7060075; doi:10.1371/journal.pone.0230201)

ctl ctl ctl CM CM CM x x

98 kda-  
64 kda-  
50 kda-  
34 kda-  
30 kda -

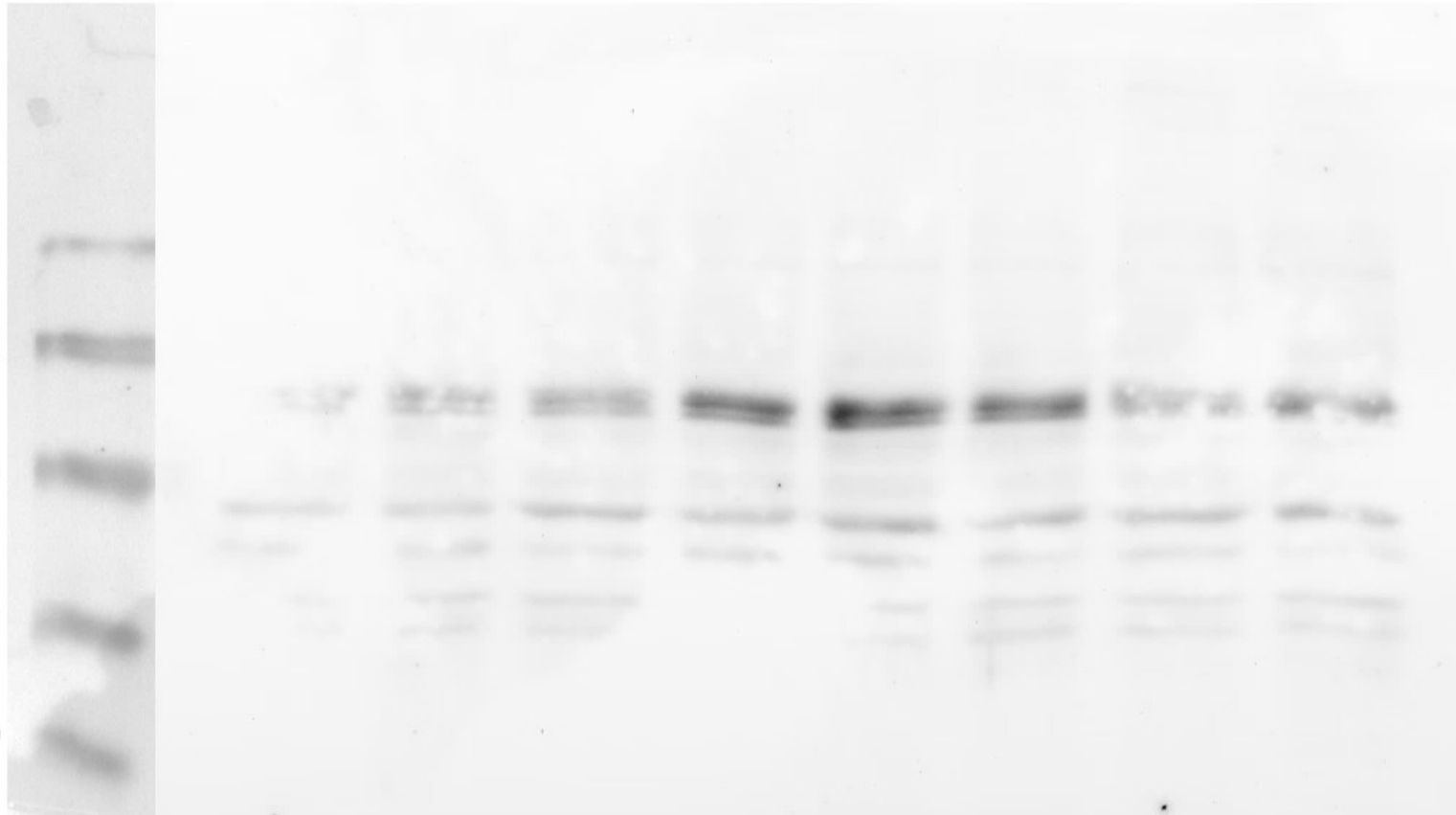

- 60

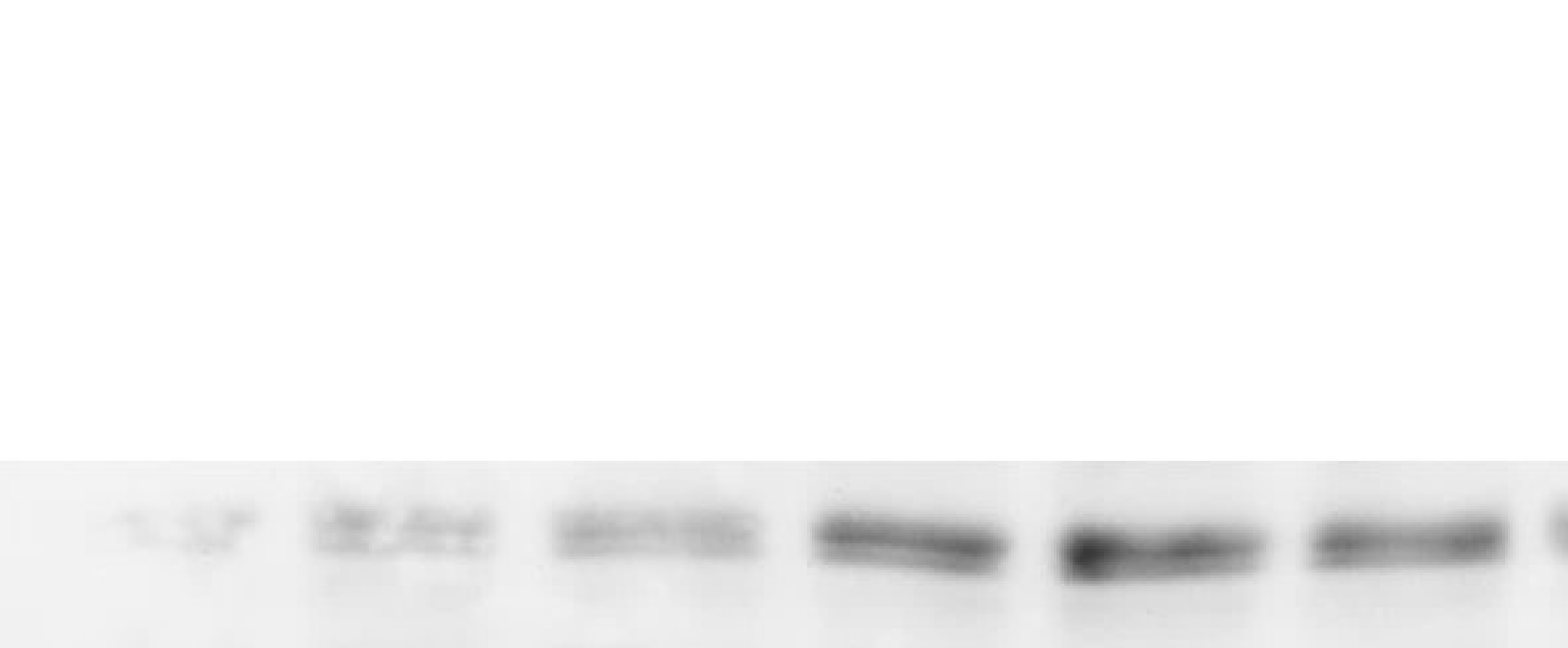

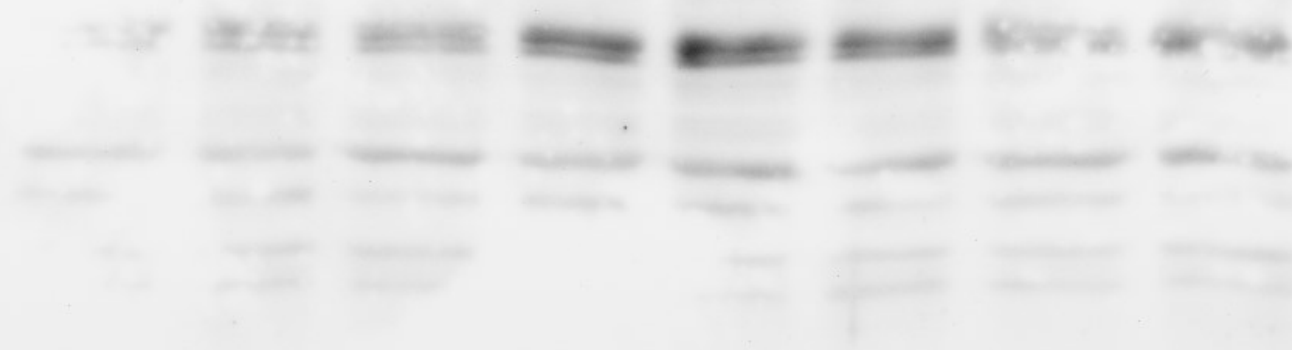



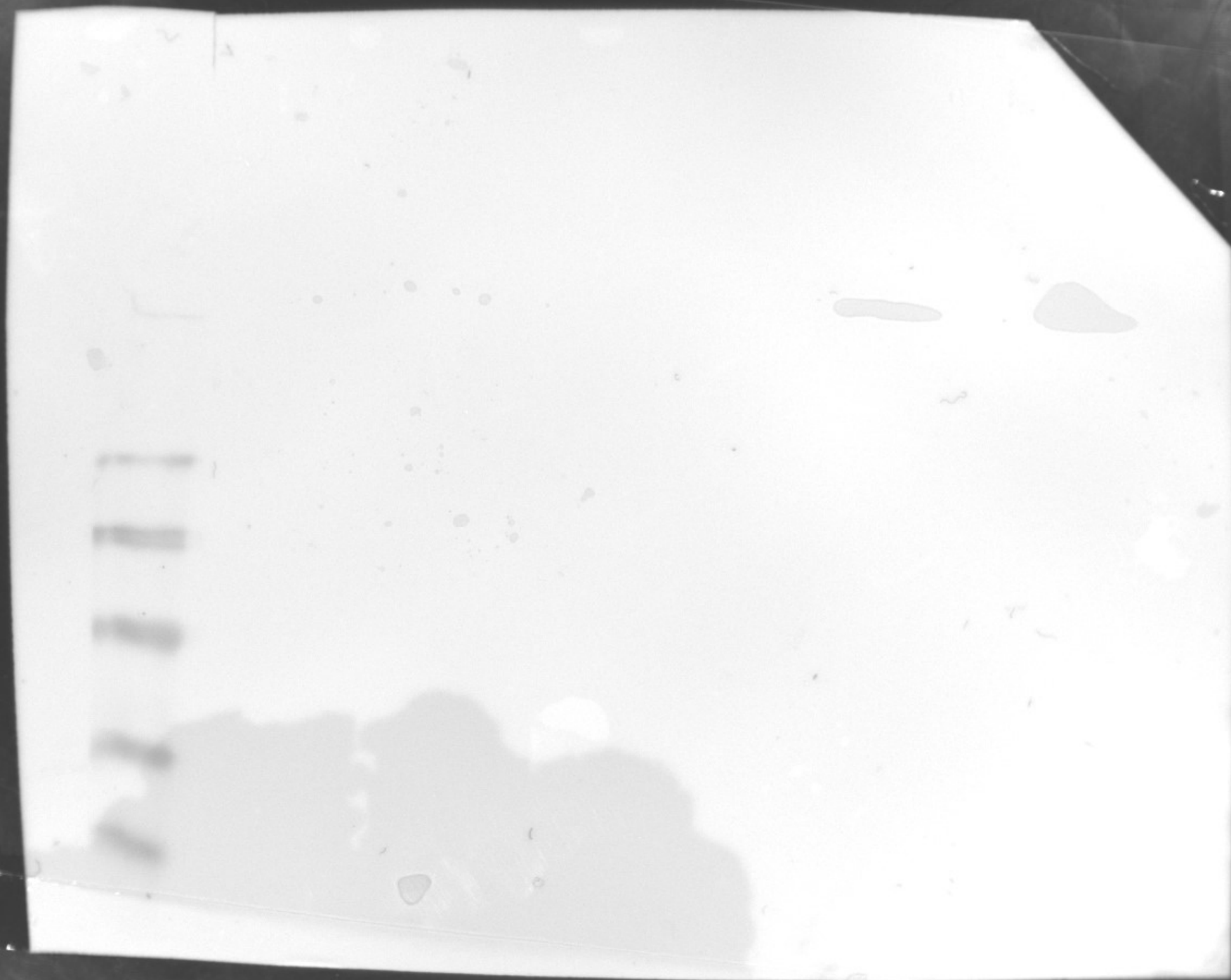

Supplement: S1 Fig — (PDF) [file pone.0230201.s005.pdf]

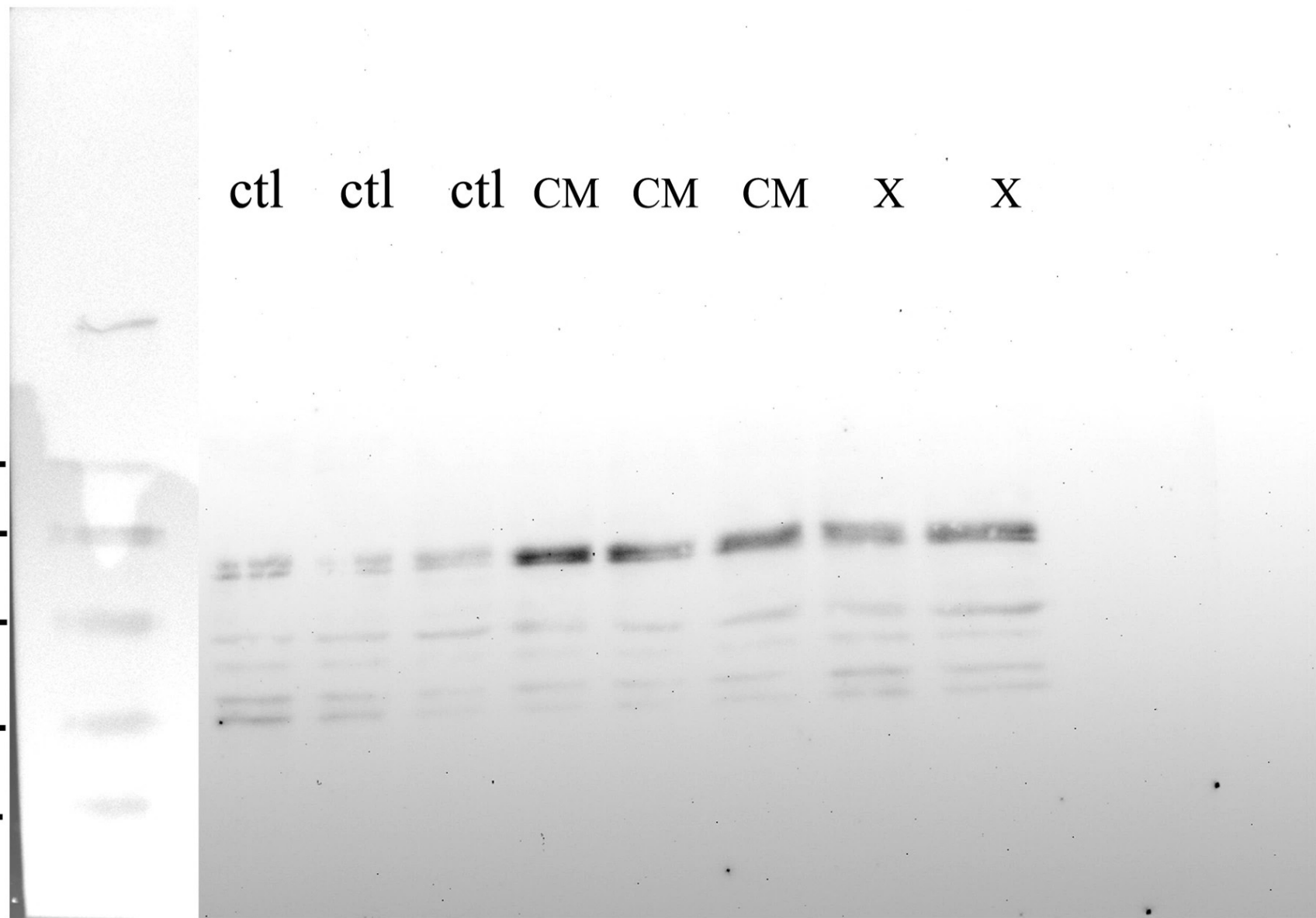

ctl    ctl    ctl    CM    CM    CM    X    X

98 kda -  
64 kda -  
50 kda -  
36 kda -  
30 kda -

- 60



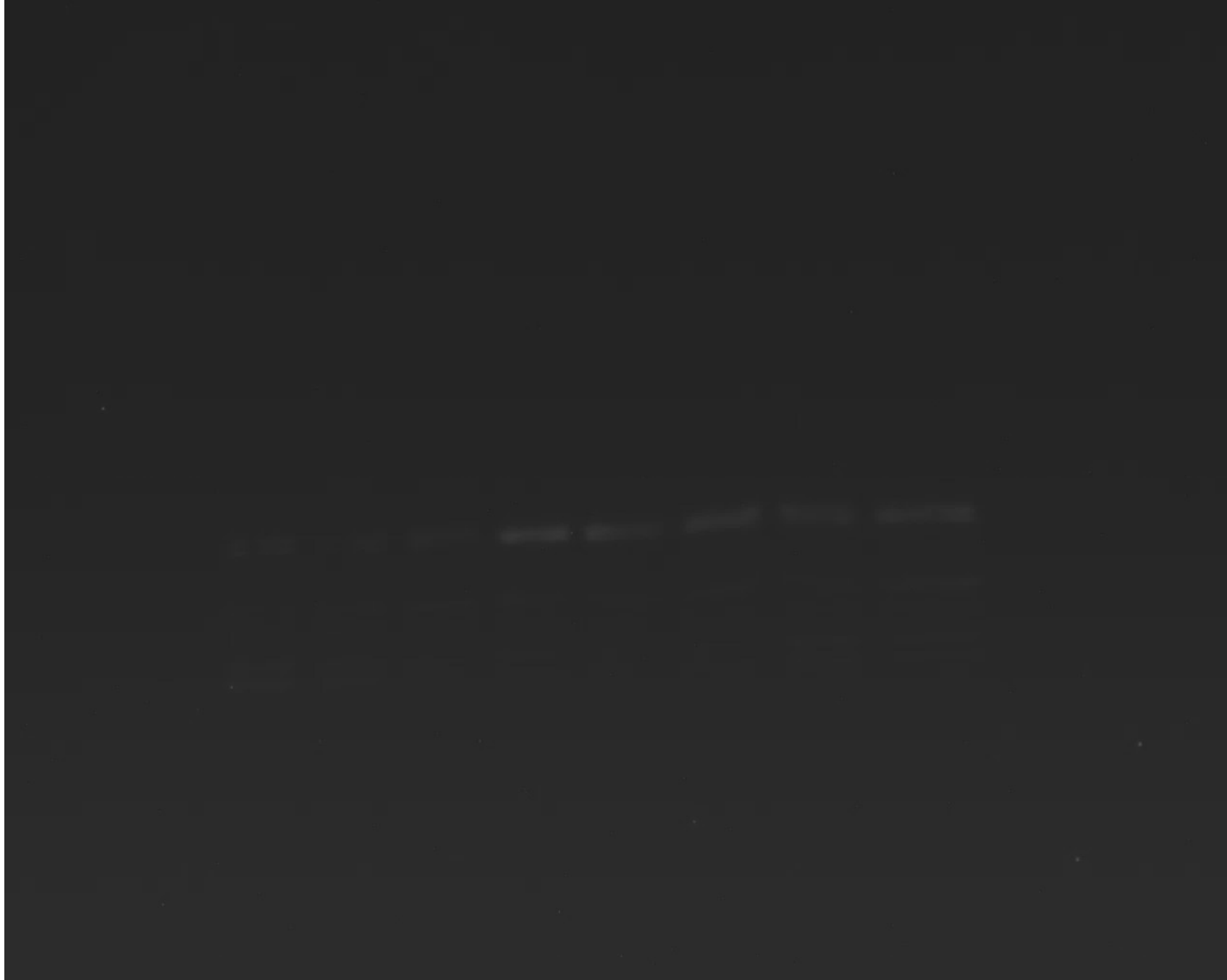

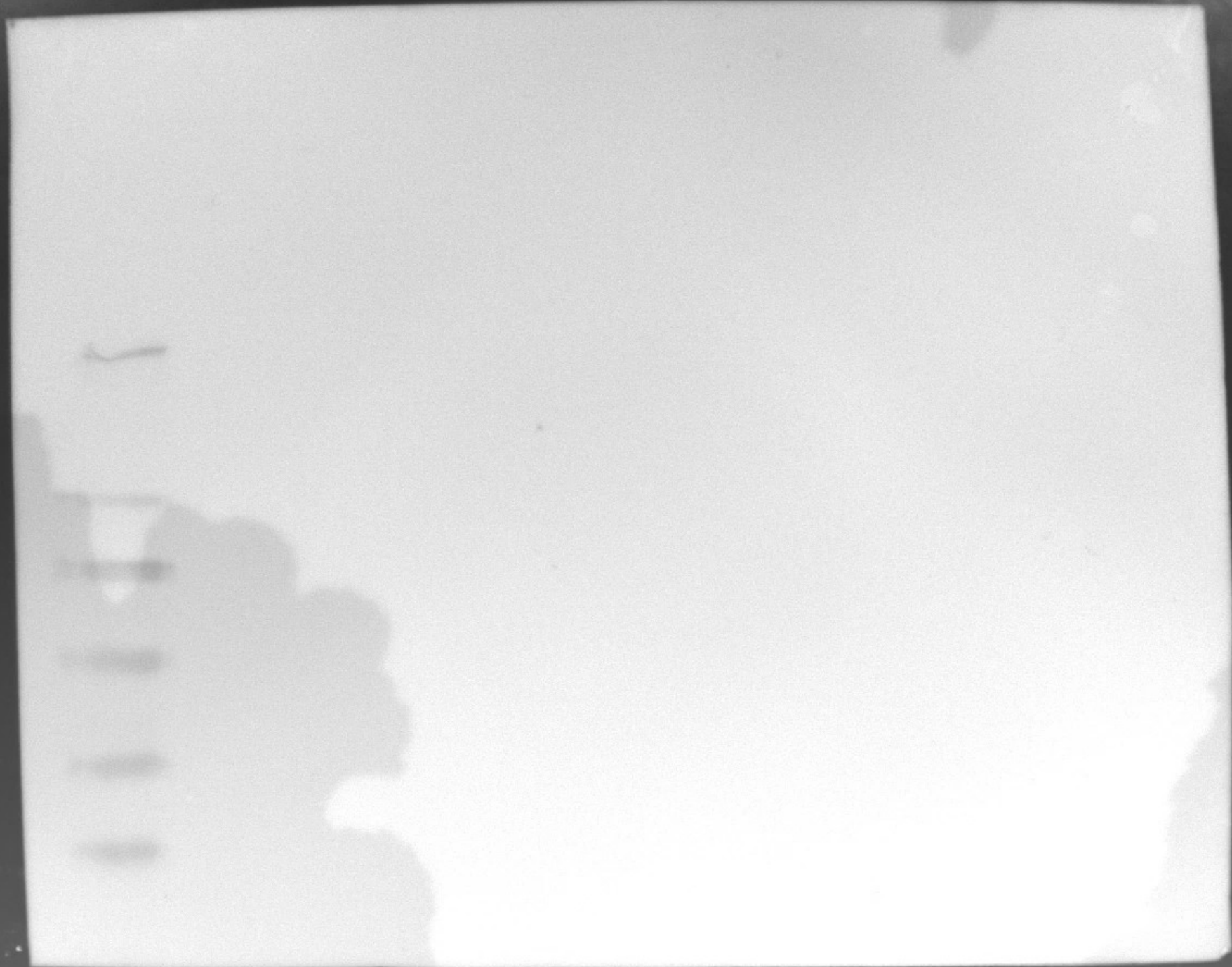

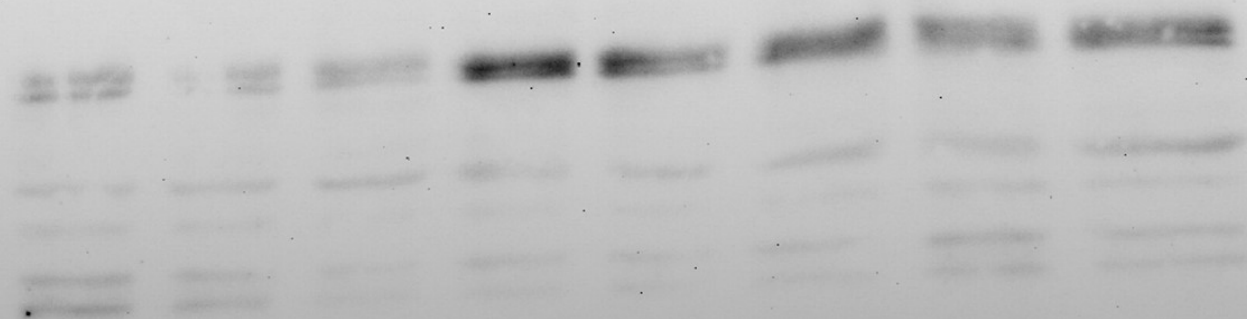

Supplement: S2 Fig — (PDF) [file pone.0230201.s006.pdf]
